# The effect of melanin-free extract from *Sepia esculenta* ink on lipid peroxidation, protein oxidation and water-holding capacity of tilapia fillet during cold storage

**DOI:** 10.1186/s13065-018-0402-9

**Published:** 2018-03-14

**Authors:** Zhen-Hua Duan, Hua-Zhong Liu, Ping Luo, Yi-Peng Gu, Yan-Qun Li

**Affiliations:** 1 Institute of Food Science & Engineering Technology, Hezhou University, Hezhou, 542899 China; 2 College of Chemistry & Environment, Guangdong Ocean University, Zhanjiang, 524088 China; 3 College of Food Science & Technology, Guangdong Ocean University, Zhanjiang, 524088 China

**Keywords:** Antioxidation, Cold storage, *Sepia esculenta* ink, Tilapia fillets

## Abstract

**Background:**

Preservative effect of melanin-free extract of *Sepia esculenta* ink (MFESI) on *Sparus latus* fillet has been verified in our previous work. This study aims to further approach the mechanism of MFESI for extending the shelf-life of fish fillet during cold storage. Tilapia fillets were treated with different dosage of MFESI (0, 15, 25 and 35 mg/ml) and packed with preservative film for succedent cold-storage at 4 °C for scheduled time. Contents of total volatile basic nitrogen and sulfydryl and carbanyl groups were measured for evaluating protein oxidation. Malondialdehyde contents were measured for estimating lipid peroxidation and loss of water was used to determine water-holding capacity of fillet.

**Results:**

The data indicated that MFESI not only possessed certain degree of antioxidant capacity in vitro, also lengthened shelf-life of tilapia fillet in cold-storage condition. Apart from 15 mg/ml, both 25 and 35 mg/ml of MFESI obviously prevented lipid and protein from oxidation and reduced loss of water from tilapia fillets, and the latter was more effective than the former.

**Conclusion:**

MFESI can repress lipid peroxidation and protein oxidation and reduce water loss, maintain the tilapia fillets quality and, thus, it could be an effective and natural preservative for extending the shelf-life of tilapia fillets during cold storage.

## Background

As a delicious food and a good resource of proteins in human diet, fish plays an important role in the global food supply. However, fish is difficult to keep fresh and even highly perishable due to the actions of microorganisms and enzymes naturally present and rancidity of the lipids. In order to keep the quality of fish, a lot of techniques to reduce the deterioration have been developed. Although the chemical preservatives are efficient and cheap, their health hazards are the concerns of consumers and regulations and the addition of synthetic preservatives has been restricted. Nowadays, the applications of safe and natural-source preservatives in the fish processing are still limited. Therefore, it is an urgent task to develop efficient, safe and natural preservatives for fish processing industry.

Sepia ink is a marine material with multifunctional roles based on its bioactive components, including protein, melanin and glycosaminoglycan [1]. Regrettably sepia ink is generally discarded during the fish process. To fully utilize the by-product of squid processing, attempts have been made by researchers. The potential fresh-keeping effects of sepia ink were approached in shiokara and peeled shrimp in earlier studies [2–6]. Similarly, our previous work also revealed the fresh-keeping effects of sepia ink. A melanin-free extract from sepia ink (MFESI) had demonstrated a capacity for significant prolongation of shelf-life on *Sparus latus* fillet and its preservative effect was revealed to be correlated with the suppression of oxidation and the spoilage microorganisms [7–9].

Tilapia is an economic and globally important aquaculture food commodity [10]. In 2015, the world aquaculture production of tilapia amounted 5,670,981 t (FAO, 2017). For this reason, tilapia was selected as experimental material in this research for investigation of the preservative mechanisms of sepia ink extract and its fresh-keeping effects on freshwater fish during cold storage, through comprehensive evaluations on lipid peroxidation, protein oxidation and water holding capacity in tilapia fillets.

## Methods

### Preparation of melanin-free extract from sepia ink

The extracting procedure was modified slightly according to our reported methods [8] and described as follows. Fresh ink taken from cuttlefish sacs (*Sepia esculenta*) was stored at − 70 °C for subsequent use. Before extraction, the frozen ink was thawed at 4 °C followed by dilution with phosphate buffered solution (PBS, pH 7.2) and sonication. The mixture was stored at 4 °C for more than 8 h and then was subjected to be centrifuged at 4 °C, 8000 rpm for 50 min. Supernatant was centrifuged for three times and then was harvested to be heated in 50 °C water bath for 1 h. The melanin-free extract was dialyzed to remove chemicals and was concentrated in turn with rotary evaporator. The concentrated extract was determined to be 35 mg/ml (high concentration, H) using drying method, and was then diluted to the other different concentrations with distilled water, 25 mg/ml (middle concentration, M) and 15 mg/ml (low concentration, L).

### Sampling and treatment

Fresh tilapias (purchased from local aquaculture market in Zhanjiang, China) were sacrificed and the ridge meat was used to prepare fillets (1 cm × 2 cm × 3 cm). Fillets were washed with ice-cold normal saline and were then immersed in different concentrations of MFESI for 5 min respectively (m/v, 1/3). Drained fillets were packed with preservative film and were stored at 4 °C for the following determination.

### Antioxidant capacity assay

Scavenging activity of hydroxyl free or DPPH (1,1-diphenyl-2-picrylhydrazyl) radical was determined according to the previously described methods [11].

DPPH radical: 2 ml of DPPH solution (0.1 mmol/l) was mixed with 0.5 ml of MFESI (35 mg/ml) and 1.5 ml H_2_O, and was then kept for 30 min at ambient temperature. Optical density value was read at 517 nm.

$$Scavenging\;activity\;\; (\% ) = \frac{{1 - (OD_{2} - OD_{1} )}}{{OD_{0} }} \times 100\%$$

OD_0_: DPPH, ethanol; OD_1_: ethanol, MFESI and water; OD_2_: DPPH-ethanol, MFESI and water.

Hydroxyl free radical: 1 ml of sample solution (0.125–1 mg/ml) in PBS (0.02 mol/l, pH 7.4) was mixed with 1.5 ml of 1,10-phenanthroline (1 mmol/l), 1 ml of FeSO_4_ (1.5 mmol/l), 1 ml of H_2_O_2_ (1%) and 3.5 ml of ultra pure water. After incubation for 60 min at 37 °C, optical density was read at 536 nm. Scavenging rate (%) was calculated according to the formula.

$$Scavenging\;activity \;\;(\% ) = \frac{{OD_{2} - OD_{1} }}{{OD_{0} - OD_{1} }} \times 100\%$$

OD_1_: no sample; OD_0_: no sample and H_2_O_2_; OD_2_: sample.

### Biochemical assay

Total volatile basic nitrogen (TVB-N) content was determined according to the previously described method [12]. Contents of sulfhydryl group, carbanyl group and malondialdehyde (MDA) were measured with detection kits developed by a bioengineering institute in China according to manufacturer’s protocol.

### Water-holding capacity

Water-holding capacity (WHC) was determined with the method of Lakshmanan et al. [13] that was slightly modified and described briefly as follows. Two grams of fish mince was placed into Eppendorf tube that has been placed in two pieces of filter paper and been weighed. Tube was centrifuged at 10 °C, 3000 rpm for 10 min, and then filter paper was weighed again. WHC (%) of fish meat was expressed as: 1 – 100% × (m_2_ − m_1_)/m.

m_2_: quality of centrifuged filter paper; m_1_: quality of uncentrifuged filter paper; m: quality of uncentrifuged fish meat (2.00 ± 0.01).

### Data analysis

Data were expressed as the mean ± standard deviation. Differences between groups were analyzed by one-way ANOVA using the JMP statistical software. *p* < 0.05 was considered to be significant level.

## Results

### In vitro antioxidant capacity of MFESI

Antioxidant capacity of MFESI was determined in vitro, and the result showed that, when the dosage of MFESI was 35 mg/ml, the scavenging rate of hydroxyl free radical and DPPH radical were 25.77 and 32.64%, respectively (Table 1).

**Table 1:** In vitro antioxidant capacity of MFESI (35 mg/ml, n = 5)

	Antioxidant capicity
Scavenging activity of hydroxyl free radical (%)	25.77 ± 1.30
Scavenging activity of DPPH radical (%)	32.64 ± 2.09

### TVB-N in fillet was reduced by MFESI

Total volatile basic nitrogen (TVB-N) of tilapia fillets was observed under the treatments with MFESI in different dosages along the experimental proceeding time. The results showed that the TVB-N values increased with the prolongation of proceeding time and raising of dosage (Table 2). However, in all of the observed samples, no significant decrease of TVB-N value was found within 48 h, while there was an obvious reduction of TVB-N in high dosage after 48 h.

**Table 2:** Inhibition of TVBN production by MFESI in fillets

Group	0 h	24 h	48 h	72 h	96 h	120 h	144 h
Vehicle	3.78 ± 0.67^a^	5.67 ± 0.32^a^	13.23 ± 0.84^a^*	16.80 ± 1.03^a^*	18.90 ± 0.99^a^*	23.80 ± 0.84^a^*	26.13 ± 1.12^a^*
L	3.36 ± 0.39^a^	5.67 ± 0.70^a^	11.97 ± 0.89^ab^*	16.33 ± 0.81^a^*	17.73 ± 1.71^a^*	24.27 ± 1.17^a^*	25.20 ± 0.56^ab^*
M	3.36 ± 0.42^a^	5.04 ± 0.36^a^	10.08 ± 0.71^b^*	15.40 ± 1.98^a^*	17.27 ± 1.82^a^*	20.07 ± 0.81^b^*	23.33 ± 1.17^b^*
H	3.78 ± 0.76^a^	4.41 ± 0.79^a^	7.14 ± 0.89^c^*	10.27 ± 1.62^b^*	9.80 ± 1.19^b^*	14.00 ± 0.79^c^*	17.27 ± 1.14^c^*

Data represent the mean ± SD, n = 10. Vehicle, L, M and H express that the fillets has been treated with 0, 15, 25 and 35 mg/ml of MFESI, respectively. Different letters indicate significant between-group differences, ^abc^ *p* < 0.05. Asterisk expresses significant intra-group differences compared to the treated sample with MFESI for 0 h, * *p* < 0.05

### Lipid peroxidation in fillet was suppressed by MFESI

Data in Table 3 shows increased MDA contents in the fillets treated with MFESI. In all of treated fillets, MDA contents after 48 h of the treating time were higher than that within the first 2 days. From 96 h, significant differences were also observed between fillets treated with vehicle and MFESI, especially with the high dosage of MFESI, suggesting that the inhibition of lipid peroxidation by MFESI occurred in the fillets.

**Table 3:** Inhibition of MDA production by MFESI in fillets

Group	0 h	24 h	48 h	72 h	96 h	120 h	144 h
Vehicle	0.10 ± 0.02^a^	0.15 ± 0.02^a^	0.39 ± 0.03^a^*	0.41 ± 0.03^a^*	0.75 ± 0.02^a^*	0.91 ± 0.05^a^*	0.94 ± 0.05^a^*
L	0.09 ± 0.04^a^	0.14 ± 0.03^a^	0.36 ± 0.04^a^*	0.41 ± 0.02^a^*	0.69 ± 0.06^ab^*	0.65 ± 0.05^b^*	0.67 ± 0.02^b^*
M	0.09 ± 0.05^a^	0.13 ± 0.02^a^	0.38 ± 0.03^a^*	0.38 ± 0.02^a^*	0.59 ± 0.07^b^*	0.57 ± 0.03^b^*	0.66 ± 0.04^ab^*
H	0.09 ± 0.03^a^	0.11 ± 0.04^a^	0.37 ± 0.03^a^*	0.39 ± 0.06^a^*	0.39 ± 0.03^c^*	0.41 ± 0.05^c^*	0.45 ± 0.05^b^*

Data represent the mean ± SD, n = 10. Vehicle, L, M and H express that the fillets has been treated with 0, 15, 25 and 35 mg/ml of MFESI, respectively. Different letters indicate significant between-group differences, ^abc^ *p* < 0.05. Asterisk expresses significant intra-group differences compared to the treated sample with MFESI for 0 h, * *p* < 0.05

### Protein oxidation in fillet was inhibited by MFESI

Formation of carbonyl compounds was employed to estimate the protein oxidation of the fillets in the current study. As the results shown in Table 4, tilapia fillets treated with MFESI exhibited significantly lower carbonyl contents than those treated with only vehicle, and the reductions of the carbonyl contents were more obvious at the higher MFESI concentrations. The carbonyl contents in fillets of each group were observed increasing gradually with the time, whilst the incremental degrees were remarkably different between the groups of fillets. Carbonyl contents in fillets treated with vehicle and low dosage of MFESI pronouncedly increased from 24 h of treating time, whereas the increases of carbonyl content in middle and high dosage groups were only visible after 48 and 96 h, respectively.

**Table 4:** Inhibition of carbanyl group production by MFESI in fillets

Group	0 h	24 h	48 h	72 h	96 h	120 h	144 h
Vehicle	0.38 ± 0.03^a^	0.71 ± 0.03^a^*	0.82 ± 0.06^a^*	1.01 ± 0.02^a^*	1.17 ± 0.06^a^*	1.61 ± 0.09^a^*	2.11 ± 0.09^a^*
L	0.35 ± 0.04^a^	0.75 ± 0.06^a^*	0.76 ± 0.03^a^*	1.03 ± 0.11^a^*	1.33 ± 0.07^a^*	1.49 ± 0.10^ab^*	1.85 ± 0.09^b^*
M	0.36 ± 0.05^a^	0.56 ± 0.09^ab^	0.75 ± 0.07^a^*	0.78 ± 0.07^b^*	0.80 ± 0.05^b^*	1.27 ± 0.06^b^*	1.36 ± 0.08^c^*
H	0.35 ± 0.06^a^	0.45 ± 0.07^b^	0.52 ± 0.04^b^	0.58 ± 0.06^c^	0.70 ± 0.05^b^*	0.73 ± 0.08^c^*	0.95 ± 0.05^d^*

Data represent the mean ± SD, n = 10. Vehicle, L, M and H express that the fillets has been treated with 0, 15, 25 and 35 mg/ml of MFESI, respectively. Different letters indicate significant between-group differences, ^abcd^ *p* < 0.05. Asterisk expresses significant intra-group differences compared to the treated sample with MFESI for 0 h, * *p* < 0.05

The changes of total and protein sulphydryl group contents were also observed (Tables 5 and 6). The results showed that the total and protein sulphydryl group contents were found decreased with treating time. The reductions, however, were effectively retarded by MFESI in a dose-dependent manner from 24 h (vehicle and low dosage), 48 h (middle dosage) and 96 h (high dosage), respectively.

**Table 5:** Inhibition of protein sulfhydryl group reduction by MFESI in fillets

Group	0 h	24 h	48 h	72 h	96 h	120 h	144 h
Vehicle	16.52 ± 0.41^a^	14.43 ± 0.38^a^*	12.94 ± 0.17^a^*	12.05 ± 0.44^a^*	9.86 ± 0.55^a^*	8.43 ± 0.48^a^*	9.60 ± 0.48^a^*
L	16.76 ± 0.27^a^	14.86 ± 0.20^a^*	13.35 ± 0.78^a^*	13.01 ± 0.84^a^*	12.14 ± 0.41^b^*	10.60 ± 0.69^b^*	10.21 ± 0.23^a^*
M	16.59 ± 0.58^a^	15.88 ± 0.41^ab^	15.11 ± 0.68^b^*	14.05 ± 0.32^ab^*	12.99 ± 0.59^b^*	12.74 ± 0.59^c^*	12.37 ± 0.74^b^*
H	16.51 ± 0.37^a^	16.27 ± 0.52^b^	15.98 ± 0.84^b^	15.37 ± 0.56^b^	14.89 ± 0.20^c^*	14.41 ± 0.61^d^*	14.51 ± 0.66^c^*

Data represent the mean ± SD, n = 10. Vehicle, L, M and H express that the fillets has been treated with 0, 15, 25 and 35 mg/ml of MFESI, respectively. Different letters indicate significant between-group differences, ^abcd^ *p* < 0.05. Asterisk expresses significant intra-group differences compared to the treated sample with MFESI for 0 h, * *p* < 0.05

**Table 6:** Inhibition of total sulfhydryl group reduction by MFESI in fillets

Group	0 h	24 h	48 h	72 h	96 h	120 h	144 h
vehicle	18.03 ± 0.54^a^	15.39 ± 0.32^a^*	14.95 ± 0.78^a^*	14.44 ± 0.73^a^*	14.21 ± 0.60^a^*	11.36 ± 0.67^a^*	12.26 ± 0.54^a^*
L	18.33 ± 0.56^a^	15.88 ± 0.34^ab^*	15.10 ± 0.08^ab^*	14.92 ± 0.91^a^*	14.41 ± 0.76^a^*	13.57 ± 0.84^b^*	13.01 ± 0.77^a^*
M	18.53 ± 0.72^a^	17.37 ± 0.64^bc^	16.96 ± 0.50^bc^*	15.47 ± 0.29^a^*	14.58 ± 0.36^a^*	13.91 ± 0.29^b^*	13.08 ± 0.27^a^*
H	18.22 ± 0.42^a^	17.94 ± 0.25^c^	17.14 ± 0.84^c^	16.59 ± 0.37^b^	16.18 ± 0.40^b^*	15.65 ± 0.41^c^*	14.89 ± 0.29^b^*

Data represent the mean ± SD, n = 10. Vehicle, L, M and H express that the fillets has been treated with 0 mg/ml, 15 mg/ml, 25 mg/ml and 35 mg/ml of MFESI, respectively. Different letters indicate significant between-group differences, ^abc^ *p* < 0.05. Asterisk expresses significant intra-group differences compared to the treated sample with MFESI for 0 h, * *p* < 0.05

### Loss of water-holding capacity was prevented in MFESI-treated fillet

**Fig. 1:**
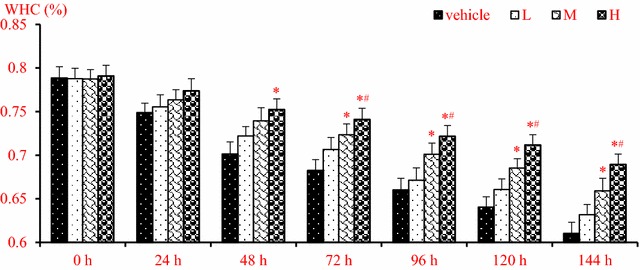
Loss of water-holding capacity of fillet was repressed by MFESI. Vehicle, L, M and H express that the fillets has been treated with 0, 15, 25 and 35 mg/ml of MFESI, respectively. *p < 0.05 expresses the difference compared to vehicle treated fillet; ^#^*p* < 0.05, vs low dosage of MFESI (15 mg/ml) treated fillet

WHCs of fillets treated with vehicle and different doses of MFESI (L, M and H) were determined, respectively, as shown in Fig. 1. The results exhibited an improvement of WHC along with the dosage increase of MFESI (*p* < 0.05). In comparison with vehicle, high dosage of MFESI harvested the most effective protection on WHC demonstrated by data at 48 h (*p* < 0.05) and the later treating time (*p* < 0.05). However, the visible difference was dedicated from 72 h (*p* < 0.05) in middle dosage of MFESI. Moreover, low dosage of MFESI failed to rescue the WHC decline. In addition, it can be found that there was more increase of WHC in the high dose treatment group than that in the middle dose group from 96 h.

### Correlation among the indicators

In order to reveal relationships among the detected variables in tilapia fillets treated with high dosage of MFESI, Pearson correlation coefficients were analyzed and showed in Table 7. The results indicated strong relationships (*p* < 0.01) among lipid peroxidation, protein oxidation and water-holding capacity in MFESI-treated tilapia fillet during cold storage.

**Table 7:** Pearson correlation coefficients between measured variables

	MDA	Protein sulfhydryl	Carbanyl group	WHC
TVBN	0.85**	− 0.94**	0.96**	− 0.97**
MDA		− 0.85**	0.82**	− 0.89**
Protein sulfhydryl			− 0.93**	0.97**
Carbanyl group				− 0.98**

Asterisk expresses significant difference between the two intersection indicators, **p < 0.01

## Discussion

Sepia ink has been proved to be a multifunctional marine material containing melanin, lipid, protein, polysaccharide and microelements [14]. The sepia ink polysaccharides (SIP) from *Sepia esculenta* ink is categorized as glycosaminoglycan mainly consisted of arabinose and aminogalactose [15]. MFESI and SIP have been proved to have antioxidant activity by our previous work based on in vivo and in vitro investigations, such as scavenging hydroxyl free radical and DPPH radical, preventing DNA from damage induced by H_2_O_2_ and ultraviolet radiation [16–19]. DPPH is a synthetic, stable free-radical containing three benzene rings and a lone electron in a nitrogen atom [20]. Aubergine DPPH captures a hydrogen atom from antioxidant to form yellow unfree DPPH-H [21]. In MFESI solution, many constituents, including polysaccharide, protein, lipid and melanin, can provide hydrogen. Consequently, DPPH was deleted by MFESI. And, higher concentration of MFESI provided more hydrogen, so antioxidant capacity increased with rising concentration of MFESI.

Hydroxyl radical is the most active one of reactive oxide species and reacts with biological macromolecules, such as protein, lipid and DNA through hydrogen abstraction, addition and electron transfer mechanisms [22]. We previously found that DNA breakage induced by hydroxyl originated from H_2_O_2_ exposed to UV can be prevented by SIP via inhibiting the activation of H_2_O_2_ by UV [17]. In this study, with the addition of MFESI into the Fenton reaction system, the reduction of hydroxyl radical content might correlate with suppression of Fenton reaction. However the accurate mechanism should be explained in the following work.

It is well known that oxidants, such as radicals, can lead to destruction of protein and lipid, resulting in cytolysis, which is a critical cause for shortening shelf-life of preserved food, especially fishes with large amount of polyunsaturated fatty acids. Our report revealed fresh-keeping effect of MFESI on marine fish demonstrated by elongated shelf-life that resulted from inhibition of bacteria growth and protein degradation [8].

Total volatile basic nitrogen (TVBN) is a group of nitrogen-containing compounds, including NH_3_ and amines, originated from protein degradation by enzymes and bacteria [23]. This study showed significant reduction of TVBN by MFESI in tilapia fillet during cold storage, which could be explained by the following mechanisms. Firstly, inhibition of bacteria by MFESI blocked protein degradation [8]. Secondly, SIP activated Nrf2/ARE pathway, an important antioxidation-associated signaling pathway, to delete oxidants [24]. Thirdly, SIP can prevent effectively cells from oxidants induced autophagy, ameliorating formation of autophagosome [15, 25]. Therefore, in our current research, a possible mechanism was that the liberation of hydrolases from lysosomes was inhibited by SIP, so that the degradation of protein and the formation of TVBN were weakened.

Apart from TVBN, two indicators of protein disruption are loss of sulphydryl group and production of carbanyl group. Determination of carbonyl is considered as a routine procedure for evaluating protein oxidation, but it is not very accurate to estimate the status of protein oxidation due to the presence of various originated carbonyls, such as derivatization agent and lipid-derived carbonyls [26]. Reactive oxygen, such as hydroxyl radical, can break peptide bond to form carbonyl [27]. Hydroxyl radicals were scavenged by MFESI, resulting in inhibition of production of carbonyl from proteins. Another indicator as a complementary technique of protein oxidation is loss of sulphydryl group of protein due to formation of disulphide bond, which partly results from lipid oxidation. NO induces nitrosation of protein sulfydryl, reducing protein sulfydryl that can be also caused by other oxidants [28]. SIP can reduce NO via activating Nrf2/ARE signaling pathway [24, 29, 30]. Therefore, MFESI decreased NO and oxygen radical contents, protecting protein from oxidation and consequently repressing increase of carbonyl and decrease of sulfydryl.

Additionally, lipid peroxidation is both a promoter of protein oxidation and another important cause of reducing quality and shelf-life of meats. *Sepia* ink and SIP possess antioxidant activities [16–19, 24, 31], which prevents lipid from oxidants mediated peroxidation [22]. As a secondary product and an indicator of lipid peroxidation, MDA content in fillet expresses degree of lipid oxidation. This study revealed that MFESI definitely inhibited lipid peroxidation in fillets, and the inhibition increased with the extract concentration.

Lipid oxidative products lead protein to oxidation degradation. Also, lipid oxidation and protein oxidation occur independently or parallel [32, 33]. Combining the data of lipid peroxidation and protein oxidation in tilapia fillets during cold storage, two important topics can be deduced reasonably. One is that MFESI definitely inhibited oxidation of lipid and protein. Another topic is that lipid peroxidation and protein oxidation occurred independently in the beginning stage (before 48 h). In the fillets treated with vehicle or low-dosage MFESI, protein oxidation was visible at 24 h whilst lipid peroxidation products were found at 48 h. Apparently, protein oxidation occurred before lipid peroxidation.

Aside from carbonyl formation and sulfhydryl reduction, protein oxidation brings about another outcome, alternation of water holding capacity (WHC). WHC expresses the capacity of muscle resisting water loss. There are two types of water forms, free and bound, accounting for 90 and 10%, respectively, in fish tissues. Free water can be influenced by various factors, such as protein structure and pH, and so on [13, 34]. Protein determines distribution of water in meat, affecting directly WHC of meat. Increase of WHC indicated that protein degradation was suppressed by MFESI during the cold-storage of fillet.

To further understand relationship among lipid peroxidation, protein oxidation and water-holding capacity, Pearson correlation was analyzed among all of the measured indicators in high dosage of MFESI treated fillets. The results indicated strong relationships among lipid peroxidation, protein oxidation and water-holding capacity in MFESI-administered tilapia fillet during cold storage.

Summarily, based on our previous findings about fresh-keeping effects of MFESI on marine fish, this study further investigated the involved mechanisms on freshwater fish through assessment of oxidation of lipid and protein, as well as WHC of fillets. Results revealed that MFESI prevented effectively fillets from protein oxidation and lipid peroxidation through eliminating radicals, WHC was maintained. Consequently, quality of fish meat was kept and shelf-time was extended undoubtedly. *Sepia* ink has a long history of being used in various ways in food and drugs [14], suggesting that it is edible safety. It is can be seen that MFESI is a potential natural preservative for fish and other foods.
